# Burden of Hospital Admission and Repeat Angiography in Angina Pectoris Patients with and without Coronary Artery Disease: A Registry-Based Cohort Study

**DOI:** 10.1371/journal.pone.0093170

**Published:** 2014-04-04

**Authors:** Lasse Jespersen, Steen Z. Abildstrom, Anders Hvelplund, Jan K. Madsen, Soren Galatius, Frants Pedersen, Soren Hojberg, Eva Prescott

**Affiliations:** 1 Department of Cardiology, Bispebjerg University Hospital, Copenhagen, Denmark; 2 National Institute of Public Health, University of Southern Denmark, Copenhagen, Denmark; 3 Department of Cardiology, Gentofte University Hospital, Hellerup, Denmark; 4 Department of Cardiology, Rigshospitalet University Hospital, Copenhagen, Denmark; 5 Copenhagen City Heart Study, Bispebjerg University Hospital, Copenhagen, Denmark; University Heart Center Freiburg, Germany

## Abstract

**Aims:**

To evaluate risk of hospitalization due to cardiovascular disease (CVD) and repeat coronary angiography (CAG) in stable angina pectoris (SAP) with no obstructive coronary artery disease (CAD) versus obstructive CAD, and asymptomatic reference individuals.

**Methods and Results:**

We followed 11,223 patients with no prior CVD having a first-time CAG in 1998–2009 due to SAP symptoms and 5,695 asymptomatic reference individuals from the Copenhagen City Heart Study through registry linkage for 7.8 years (median). In recurrent event survival analysis, patients with SAP had 3–4-fold higher risk of hospitalization for CVD irrespective of CAG findings and cardiovascular comorbidity. Multivariable adjusted hazard ratios(95%CI) for patients with angiographically normal coronary arteries was 3.0(2.5–3.5), for angiographically diffuse non-obstructive CAD 3.9(3.3–4.6) and for 1–3-vessel disease 3.6–4.1(range)(all P<0.001). Mean accumulated hospitalization time was 3.5(3.0–4.0)(days/10 years follow-up) in reference individuals and 4.5(3.8–5.2)/7.0(5.4–8.6)/6.7(5.2–8.1)/6.1(5.2–7.4)/8.6(6.6–10.7) in patients with angiographically normal coronary arteries/angiographically diffuse non-obstructive CAD/1-, 2-, and 3-vessel disease, respectively (all P<0.05, age-adjusted). SAP symptoms predicted repeat CAG with multivariable adjusted hazard ratios for patients with angiographically normal coronary arteries being 2.3(1.9–2.9), for angiographically diffuse non-obstructive CAD 5.5(4.4–6.8) and for obstructive CAD 6.6–9.4(range)(all P<0.001).

**Conclusions:**

Patients with SAP symptoms and angiographically normal coronary arteries or angiographically diffuse non-obstructive CAD suffer from considerably greater CVD burdens in terms of hospitalization for CVD and repeat CAG compared with asymptomatic reference individuals even after adjustment for cardiac risk factors and exclusion of cardiovascular comorbidity as cause. Contrary to common perception, excluding obstructive CAD by CAG in such patients does not ensure a benign cardiovascular prognosis.

## Introduction

Stable angina pectoris (SAP) symptoms with no obstructive coronary artery disease (CAD) at angiography remain a great challenge for physicians and patients. Not only is this seemingly paradoxical condition frequent in clinical practice, as nearly two thirds of women and one third of men undergoing first-time coronary angiography (CAG) due to symptoms of SAP are found to have no obstructive CAD (defined as 0–49% coronary artery stenosis), but it is also associated with increased risk of major cardiovascular events, and symptoms persisting for years. [1], [2] However, the previously published ‘time-to-first-event’ analyses do not fully reflect the true burden of disease from symptoms of SAP and no obstructive CAD at angiography which would require taking recurrent events into account. Additionally, previous risk analyses of SAP symptoms with no obstructive CAD at angiography ignore a vast amount of information on the patient’s outcomes such as a broader spectrum of hospital admissions, re-catheterizations, and primary care contacts. While the impact of death and major adverse cardiovascular events are obvious, the importance of softer endpoints should not be underestimated. Such events are not only distressing for patients and their families, but they may also very well be a major driver of the economic burden of SAP symptoms with no obstructive CAD at angiography. [3].

The aim of the present study was to evaluate the total disease burden in terms of cardiovascular hospitalization, repeated catheterization, non-cardiovascular hospitalization and family doctor (GP) consultation in patients with SAP symptoms and no obstructive CAD at angiography compared to asymptomatic reference individuals and obstructive CAD patients.

## Methods

### Ethics Statement

This study was approved by the Danish Data Protection Agency and the Danish National Board of Health. Written informed consent was given by all participants in the CCHS which was approved by the Ethics Committee for the area of Copenhagen i.e. “De Videnskabetiske Komiteer – Region Hovedstaden” (KF 100.2039/91). No further ethical approval was necessary according to Danish legislation.

### Patient Population

**Figure 1:**
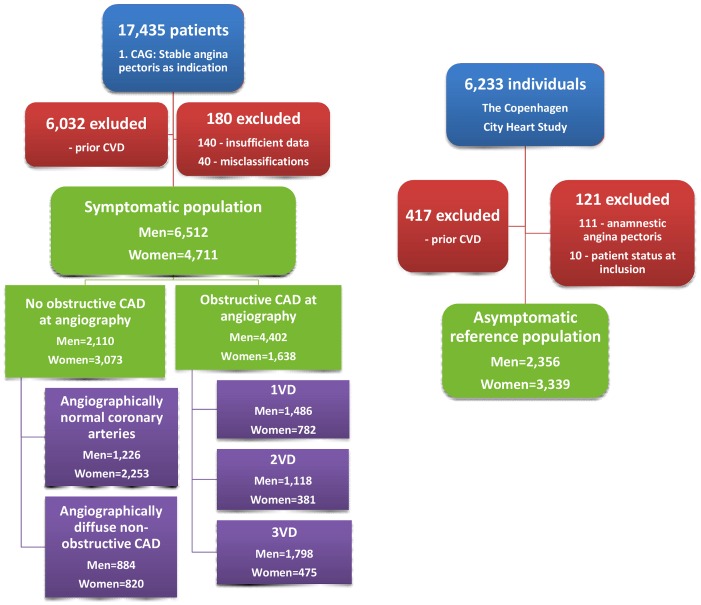
Derivation of the study population. CVD: cardiovascular disease; CAD: coronary artery disease; VD: vessel disease.

In this retrospective, registry-based cohort study, we used recurrent event survival analysis to compare risk of cardiovascular hospitalization, repeated catheterization, non-cardiovascular hospitalization and GP consultations in 11,223 patients with SAP symptoms examined with CAG and 5,695 asymptomatic, healthy individuals from the Copenhagen City Heart Study (CCHS). The patient population has previously been described in detail. [1] In summary, we identified all patients having a first-time elective CAG due to symptoms of SAP in Eastern Denmark (representing 43% of the entire Danish population) during 1998–2009 and aged 20 years or higher (n = 17,435) (Figure 1). All patients were referred to CAG by a consultant cardiologist who based this decision on the patient’s symptoms, risk factors, results of non-invasive tests and blood samples. Consequently, the SAP symptoms were defined by the decision of the cardiologist to refer the patient to CAG with SAP as indication. Patients with prior cardiovascular disease (n = 6,032) (stroke, coronary revascularization, MI or unstable angina) ascertained by central registry linkage were excluded as were patients with insufficient data (n = 140) (i.e. missing data regarding prior cardiovascular disease or degree of CAD) or misclassifications (n = 40) as explained in detail elsewhere. [1].

### Asymptomatic Reference Population

The reference population comprised individuals from the fourth part of the CCHS taking place in 2001–04. The CCHS was a prospective study initiated in 1976 with the primary goal to study the impact of lifestyle factors on cardiovascular diseases. The population was randomly selected from a certain area of approximately 90,000 residents in Copenhagen, Denmark and comprised an age-stratified sample of men and women, aged 20 years or higher. The cohort from the first examination was re-invited and supplemented by new participants from younger birth cohorts in 1981, 1991 and 2001. [4] In the present study, the reference population initially included 6,233 individuals from the fourth examination of the CCHS taking place in 2001–04 (Figure 1). Individuals with prior cardiovascular disease (n = 417) were excluded as were individuals who had typical angina (n = 111) according to WHO (Rose) Angina Questionnaire part of the CCHS, or who were already included as a patient (n = 10).

### Explanatory Variables

#### Outcome data

The primary end points included cardiovascular hospitalizations i.e. hospitalizations due to cardiovascular disease according to the primary discharge diagnosis (ICD-10: I), and repeated CAGs. The secondary end points included non-cardiovascular hospitalizations including psychiatric hospitalizations (ICD-10: not I) and GP consultations. GP consultations within the same month were registered as one.

#### Degree of coronary artery disease

The invasive cardiologists performing the baseline CAGs defined five patient groups based on the degree of CAD (Figure 1): Angiographically normal coronary arteries (no angiographic arteriosclerosis in any coronary artery), angiographically diffuse non-obstructive CAD (angiographically visible arteriosclerosis with <50% stenosis in any epicardial coronary artery), and three groups with successive degrees of obstructive CAD (i.e. ≥50% stenosis in any epicardial coronary artery): 1-(1VD), 2-(2VD) and 3-vessel disease and/or left main stem stenosis (3VD). The reference population defined a sixth group serving as comparison. The term “no obstructive CAD at angiography” designates angiographically normal coronary arteries and angiographically diffuse non-obstructive CAD as one.

#### Cardiac risk factors, comorbidity and treatment

Cardiovascular risk factors included age, sex, body mass index (BMI) (kg/m^2^), diabetes, use of antihypertensive and lipid-lowering medication, and smoking. Chest pain was classified according to the Canadian Cardiovascular Society Functional classification of angina (CCS class). Individuals were categorized as either active smokers or prior/never smokers. Body mass indexes were categorized in three groups: BMI<25, 25≤ BMI≤30 and BMI>30. Baseline comorbidity was partly based on a previous study showing that depression, migraine, rheumatic and lung diseases predict sickness absence from work in angina patients, [5] and partly on a priori assumptions regarding diseases that would potentially confound the association between the degree of CAD and outcomes. Baseline comorbidity was defined as one or more hospitalizations or outpatient contacts due to the following specific disease entities within five years pre-inclusion: cancer(ICD-10:C), migraine(ICD-10:G43), depression(ICD-10:F32–F33), chronic obstructive lung disease(COPD)(ICD-10:J44), gastroduodenal ulcer or gastroduodenitis(ICD-10:K25–27;K29), gastrooesophageal reflux(ICD-10:K20–K21;K221), bone, muscle and connective tissue diseases(ICD-10:M), and kidney diseases(ICD-10:N0–N1) and divided into three groups according to the number of diseases present: none, 1, and ≥2. Cardiac comorbidity was defined as a previous diagnosis of aortic stenosis, paroxysmal atrial fibrillation, atrial flutter, hypertrophic cardiomyopathy, or perimyocarditis or if such a diagnosis was made within 6 months post-CAG/inclusion. [1] Patients were treated medically or with angioplasty according to guidelines and physician preference. [6] We identified all claimed prescriptions of aspirin (ATC B01AC06), betablockers (ATC C07), calcium antagonists (ATC C08), angiotension converting enzyme inhibitors (ACEI) and angiotensin receptor blockers (ARBs) (ATC C09), nitrates (ATC C01D), and statins (ATC C10AA) within the first year of follow-up as well as all revascularizations. There were few missing values for all covariates (0–3%) except CCS class and left ventricular ejection fraction (missing for 14% and 53% of the symptomatic population, respectively). For further information regarding the data sources used in this study see Appendix S1.

### Statistical Analysis

Descriptive statistics were used to quantify the distribution of baseline data. The ANOVA was used to test differences in quantitative measures, and the x^2^ test was used to test differences in proportions.

Recurrent event survival analysis was performed by the Cox’s proportional hazards method using the counting process approach with robust estimation to account for the correlation among outcomes in the same subject. [7] The primary analysis aimed at comparing event rates by estimating the sex specific hazard ratio of outcome by the degree of CAD compared to asymptomatic reference individuals. The time frame in the survival analysis was defined as time from the CAG or CCHS inclusion till March 9, 2011, time of death or emigration (and thus lack of registry-based follow-up). Time during each event-associated hospitalization was not counted as time at risk, i.e. time was stopped at the time of hospitalization and restarted at discharge. In the event of transfer to other hospitals the entire hospitalization was counted as one event. Patients with obstructive CAD had higher rates of CAG and cardiovascular hospitalization during the first year of follow-up compared to the following years of follow-up. Thus, for the primary outcomes, these patients were only included in analyses concerning time from one year post-inclusion onwards. Event-free survivor functions were estimated for different groups with the Kaplan-Meier method. We used a linear regression model with robust estimation of variances (due to heteroskedasticity) to estimate mean accumulated hospitalization time per 10 years of follow-up by study population group. All initial models were age-adjusted and sex specific. Only after assessing whether the prognostic value of the degree of CAD was similar in both sexes, were the analyses repeated on pooled data and adjusted for age and cardiac risk factors. In sensitivity analyses, we further adjusted for baseline comorbidity, individuals with cardiac comorbidity were excluded and finally, we reran all Cox models as standard time-to first event analyses.

In the primary analysis, precise age-adjustment was ensured by splitting each observation in 2-year age groups above the age of 40 before entering in the model as a categorical variable. In the hospitalization time predictive model, age at time of inclusion was entered in the model as a continuous variable. We treated all other covariates as categorical variables according to the aforementioned descriptions with missing values in separate categories to avoid data loss. Since degree of CAD and risk of outcome may reflect sex specific disease characteristics, we tested for interaction with sex by testing the significance of interaction terms from regression models with the Wald test. Proportionality assumptions were tested and found valid.

All significance testing was two sided and based on a 5% probability level. All analyses were performed with the Stata 12.1 software (StataCorp, 4905 Lakeway Drive, College Station, TX, USA).

## Results

### Baseline Characteristics and Treatment within the First Year of Follow-up

Baseline characteristics of the study population are shown in Table 1 and Figure 1. Within the symptomatic population of 4,711 women and 6,512 men, a larger fraction of women (65%) than men (33%) had no obstructive CAD at angiography (P<0.001). Among women and men, correspondingly, 48 vs. 19% had angiographically normal coronary arteries and 17 vs. 14% had angiographically diffuse non-obstructive CAD.

**Table 1:** Baseline cardiac risk factors by study population group and sex.

Women	Reference	Angiogr. normal	Angiogr. diffuse	1VD	2VD	3VD
	n = 3,339	n = 2,253	n = 820	n = 782	n = 381	n = 475
**Cardiac risk factors, n(%)**						
Age(years), mean(SD)	58.9(17)	58.5(11)	65.0(10)	64.6(10)	68.0(9)	69.6(10)*
BMI(kg/m^2^), mean(SD)	25.4(5)	26.6(5)	26.9(5)	26.7(5)	26.8(5)	26.7(5)*
Diabetes	102(3)	221(10)	147(18)	135(17)	64(17)	120(25)*
Active smoking	1,038(31)	409(18)	194(24)	219(28)	82(22)	113(24)*
CCS class ≥2	–	1,086(60)	506(66)	564(80)	288(85)	391(88)*
Lipid-lowering medication	112(3)	1,062(47)	540(66)	547(70)	262(69)	341(72)*
Antihypertensive medication	598(18)	967(43)	494(60)	460(59)	224(59)	307(65)*
Comorbidity, number of						
1	795(24)	773(34)	295(36)	253(32)	130(34)	135(28)*
≥2	85(3)	132(6)	53(6)	47(6)	27(7)	17(4)
Cardiac comorbidity	20(1)	172(8)	74(9)	45(6)	28(7)	32(7)*
LVEF						
≥40	–	885(98)	335(99)	368(98)	190(94)	259(95)$
<40	–	18(2)	4(1)	7(2)	12(6)	13(5)
**Men**	**Reference**	**Angiogr. normal**	**Angiogr. diffuse**	**1VD**	**2VD**	**3VD**
	**n = 2,356**	**n = 1,226**	**n = 884**	**n = 1,486**	**n = 1,118**	**n = 1,798**
**Cardiac risk factors, n(%)**						
Age(years), mean(SD)	56.4(16)	55.9(11)	62.8(10)	61.8(10)	63.8(9)	65.3(9)*
BMI(kg/m^2^), mean(SD)	26.3(4)	27.8(5)	27.7(4)	27.4(4)	27.5(4)	27.5(4)*
Diabetes	123(5)	151(12)	189(21)	231(16)	213(19)	408(23)*
Active smoking	816(35)	343(28)	260(29)	413(28)	302(27)	462(26)*
CCS class ≥2	516(52)	456(67)	985(74)	823(80)	1,377(84)	4,157(72)*
Lipid-lowering medication	61(3)	516(42)	536(61)	963(65)	742(66)	1,286(72)*
Antihypertensive medication	299(13)	461(38)	453(51)	699(47)	563(50)	954(53)*
Comorbidity, number of						
1	416(18)	379(31)	247(28)	380(26)	275(25)	406(23)*
≥2	48(2)	64(5)	60(7)	57(4)	44(4)	58(3)
Cardiac comorbidity	17(1)	177(14)	123(14)	117(8)	87(8)	141(8)*
LVEF						
≥40	–	465(94)	334(94)	696(95)	537(93)	898(90)*
<40	–	28(6)	22(6)	36(5)	42(7)	104(10)

*P<0.001;

$ P<0.01; ^§^P≤0.05 for difference between groups (age-adjusted for all variables except age). SD: Standard deviation; BMI: body mass index; Angiogr.: angiographically. CCS class: Canadian Cardiovascular Society Functional classification of angina.

Comorbidity, i.e. cancer, migraine, depression, chronic obstructive pulmonary disease, gastroduodenal ulcer or gastroduodenitis, gastro-oesophageal reflux, bone, muscle and connective tissue diseases, and kidney diseases.

Cardiac comorbidity, i.e. aortic stenosis, atrial fibrillation/flutter, hypertrophic cardiomyopathy and perimyocarditis up to 6 months post-CAG/inclusion.

Discrepancies between counts and percentages are due to missing data.

Reference individuals and patients with angiographically normal coronary arteries were younger than patients with CAD. With the exception of smoking, cardiac risk factors and comorbidity were more prevalent in all five patient groups compared with the reference population and cardiac risk factors tended to be more prevalent with higher degrees of CAD. Table 2 shows the use of medical treatment and revascularization procedures within the first year of follow-up. Statins, aspirin and beta blockers were more frequently used in CAD patients compared with patients with angiographically normal coronary arteries and reference individuals, respectively, and medical treatment tended to be more frequent with higher degrees of CAD.

**Table 2:** Medical treatment and revascularization procedures within the first year of follow-up.

Women	Reference	Angiogr. normal	Angiogr. diffuse	1VD	2VD	3VD
	n = 3,339	n = 2,253	n = 820	n = 782	n = 381	n = 475
**Medical treatment**						
Aspirin, n(%)	341(10)	904(40)	580(71)	687(88)	333(87)	423(89)*
Beta blockers, n(%)	301(9)	854(38)	416(51)	538(69)	275(72)	365(77)*
Calcium antagonists, n(%)	315(9)	711(32)	373(45)	352(45)	179(47)	248(52)*
ACEI or ARBs, n(%)	410(12)	690(31)	383(47)	388(50)	179(47)	238(50)*
Nitrates, n(%)	61(2)	501(22)	332(40)	403(52)	234(61)	244(51)*
Statins, n(%)	176(5)	852(38)	594(72)	719(92)	347(91)	429(90)*
**Revascularisation**						
PCI, n(%)	2(0)	0(0)	5(1)	394(50)	187(49)	96(20)*
CABG, n(%)	1(0)	0(0)	0(0)	53(7)	105(28)	311(65)*
**Men**	**Reference**	**Angiogr. normal**	**Angiogr. diffuse**	**1VD**	**2VD**	**3VD**
	**n = 2,356**	**n = 1,226**	**n = 884**	**n = 1,486**	**n = 1,118**	**n = 1,798**
**Medical treatment**						
Aspirin, n(%)	206(9)	487(40)	627(71)	1,227(83)	960(86)	1,563(87)*
Beta blockers, n(%)	166(7)	468(38)	479(54)	1,024(69)	849(76)	1,385(77)*
Calcium antagonists, n(%)	178(8)	331(27)	368(42)	516(35)	486(43)	882(49)*
ACEI or ARBs, n(%)	279(12)	440(36)	442(50)	708(48)	561(50)	952(53)*
Nitrates, n(%)	40(2)	191(16)	282(32)	567(38)	523(47)	750(42)*
Statins, n(%)	118(5)	397(32)	652(74)	1,332(90)	1,017(91)	1,615(90)*
**Revascularisation**						
PCI, n(%)	5(9)	0(0)	15(2)	786(53)	548(49)	234(13)*
CABG, n(%)	2(0)	0(0)	5(1)	151(10)	362(32)	1,397(78)*

*P<0.001 for difference between groups.

### Cardiovascular Hospitalization

**Figure 2:**
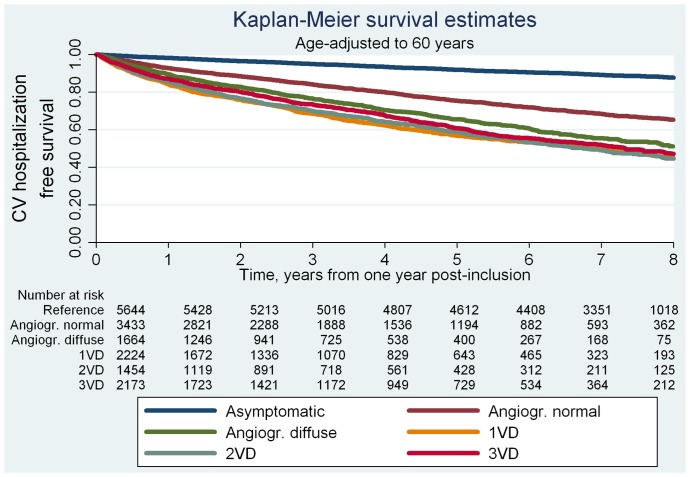
Kaplan Meier survival estimates for CV hospitalization by study population group. CV: cardiovascular; CAG: coronary angiography; Angiogr.: angiographically; VD: vessel disease.

A total of 19,870 cardiovascular hospitalizations occurred in the study population during a total follow-up of 107,446 patient years. During a median follow-up of 7.8 years (interquartile range 4.4–8.8), 2,135 women and 3,964 men were hospitalized due to cardiovascular disease. The total number of events and event rates by study population group and sex are shown in Table 3. In both men and women, patients with no obstructive CAD at angiography showed higher rates of cardiovascular hospitalization per 1000 patient years (range 107–229) than the reference population (range 36–50). We found no systematic sex differences in risk of cardiovascular hospitalization after age-adjustment (Model 1 in Table 3). Pooling men and women yielded four- to fivefold increased risks of cardiovascular hospitalization for both patient groups compared to the reference population even after multivariable adjustment. In models limited to follow-up after one-year post-inclusion, we found corresponding hazard ratios (HR)s (95%CI) for patients with angiographically normal coronary arteries of 3.0(2.5–3.5, P<0.001), angiographically diffuse non-obstructive CAD of 3.9(3.3–4.6, P<0.001), and obstructive CAD (1VD–3VD) of 3.6–4.1(range, all P<0.001). The increased risk of cardiovascular hospitalization in patients with SAP symptoms and no obstructive CAD versus reference individuals was primarily driven by increased risks of hospitalizations due to heart failure, stroke and angina (stable and unstable) in both patient groups; however, only patients with angiographically diffuse non-obstructive CAD had increased risks of hospitalization due to MI (Table S1). Accumulated hospitalization time data are shown in Table 4. Compared to the reference population’s mean accumulated hospitalization time (95%CI) of 3.5(3.0–4.0) days per 10 years of follow-up, patients with angiographically normal coronary arteries had a longer hospitalization time of 4.5(3.8–5.2, P<0.05) as did patients with angiographically diffuse non-obstructive CAD, 7.0(5.4–8.6, P<0.001) and patients with obstructive CAD 6.1–8.6(range, P<0.001). The corresponding age-adjusted event-free survival curves for cardiovascular hospitalization are shown in Figure 2. All patient groups had lower probability of age-adjusted event-free survival than the reference population. Patients with angiographically diffuse non-obstructive CAD versus obstructive CAD showed similar cardiovascular hospitalization free survival.

**Table 3:** Event rates and hazard ratios (95% confidence intervals) by end-point, study population group and sex in successively adjusted recurrent event models.

	Events; event rates per 1000 years of follow-up		Model 1^a^		Model 2^b^	Model 3^c^	Model 4^d^
End-point	Women	Men	Women	Men	Pooled data	Pooled data	Pooled data
Study population group	n = 8,050	n = 8,868	n = 8,050	n = 8,868	n = 16,918	n = 16,592	n = 10,115
**CV hospitalization**							
Reference	952; 36(32–40)	915; 50(44–56)	(Reference)	(Reference)	(Reference)	(Reference)	(Reference)
Angiographically normal	1352; 107(96–120)	1273; 189(161–222)	3.5(3.0–4.2)*	4.0(3.3–4.9)*	3.9(3.3–4.6)*	3.0(2.5–3.5)*	2.5(2.0–3.1)*
Angiographically diffuse	800; 199(171–233)	989; 229(200–263)	5.2(4.3–6.4)*	3.9(3.3–4.7)*	4.7(3.9–5.5)*	3.9(3.3–4.6)*	3.3(2.7–4.1)*
1VD	1685; 376(348–407)	3061; 357(336–380)				4.1(3.6–4.8)*	3.7(3.1–4.4)*
2VD	954; 421(381–466)	2572; 406(383–430)				3.9(3.4–4.5)*	3.5(2.9–4.2)*
3VD	1167; 418(379–462)	4296; 405(386–425)				3.6(3.1–4.1)*	3.2(2.7–3.8)*
**CAG**							
Reference	147; 6(5–7)	196; 11(9–13)	(Reference)	(Reference)	(Reference)	(Reference)	(Reference)
Angiographically normal	209; 17(14–19)	164; 24(10–29)	3.2(2.5–4.2)*	2.3(1.8–3.0)*	2.5(2.0–3.1)*	2.3(1.9–2.9)*	2.2(1.7–2.8)*
Angiographically diffuse	173; 43(36–53)	236; 55(47–64)	8.2(6.1–11.0)*	4.9(3.8–6.2)*	5.2(4.0–6.6)*	5.5(4.4–6.8)*	5.0(3.8–6.4)*
1VD	636; 142(129–156)	1020; 119(110–128)				9.4(7.7–11.4)*	8.4(6.6–10.5)*
2VD	255; 112(98–129)	708; 112(102–123)				8.1(6.6–9.9)*	6.9(5.5–8.8)*
3VD	254; 91(76–110)	829; 78(71–86)				6.6(5.3–8.1)*	6.0(4.7–7.7)*
**Non-CV hospitalization**							
Reference	6647; 250(237–264)	4566; 249(231–268)	(Reference)	(Reference)	(Reference)	(Reference)	(Reference)
Angiographically normal	4981; 396(369–426)	3005; 445(406–490)	1.9(1.7–2.0)*	2.0(1.7–2.2)*	2.0(1.8–2.1)*	1.9(1.8–2.1)*	1.9(1.7–2.1)*
Angiographically diffuse	2005; 499(448–557)	2155; 499(451–553)	2.0(1.8–2.3)*	1.9(1.7–2.1)*	2.0(1.8–2.2)*	1.9(1.7–2.1)*	1.7(1.5–1.9)*
1VD	1963; 439(397–486)	3363; 392(360–429)	1.8(1.6–2.0)*	1.5(1.3–1.7)*	1.7(1.5–1.9)*	1.6(1.5–1.8)*	1.5(1.3–1.7)*
2VD	920; 406(357–464)	2560; 404(368–444)	1.5(1.3–1.7)*	1.5(1.3–1.6)*	1.6(1.4–1.7)*	1.5(1.3–1.6)*	1.4(1.2–1.6)*
3VD	1360; 488(429–556)	4178; 394(368–422)	1.7(1.5–2.0)*	1.3(1.2–1.5)*	1.5(1.4–1.6)*	1.3(1.2–1.5)*	1.3(1.1–1.4)*
**Family doctor consultation**	(thousands)	(thousands)					
Reference	94.2; 3.6(3.5–3.6)	50.1; 2.7(2.7–2.8)	(Reference)	(Reference)	(Reference)	(Reference)	(Reference)
Angiographically normal	62.8; 5.0(4.9–5.1)	30.6; 4.6(4.4–4.7)	1.4(1.4–1.5)*	1.7(1.6–1.8)*	1.5(1.4–1.5)*	1.5(1.4–1.5)*	1.4(1.4–1.5)*
Angiographically diffuse	22.0; 5.5(5.3–5.7)	21.4; 5.0(4.8–5.2)	1.5(1.4–1.5)*	1.7(1.6–1.7)*	1.5(1.4–1.5)*	1.5(1.4–1.5)*	1.4(1.3–1.4)*
1VD	23.8; 5.3(5.2–5.5)	38.6; 4.5(4.4–4.6)	1.4(1.4–1.5)*	1.5(1.5–1.6)*	1.4(1.4–1.5)*	1.4(1.4–1.4)*	1.4(1.3–1.4)*
2VD	11.8; 5.2(5.0–5.5)	29.0; 4.6(4.4–4.7)	1.3(1.3–1.4)*	1.5(1.4–1.6)*	1.4(1.3–1.4)*	1.3(1.3–1.4)*	1.3(1.2–1.3)*
3VD	14.5; 5.2(5.0–5.4)	49.6; 4.7(4.6–4.8)	1.3(1.3–1.4)*	1.5(1.4–1.5)*	1.3(1.3–1.4)*	1.3(1.3–1.3)*	1.3(1.2–1.3)*

CV: cardiovascular disease. VD: vessel disease.

a Adjusted for age.

b Adjusted for age, body mass index, diabetes, active smoking, use of antihypertensive medication and lipid lowering medication, respectively and stratified by sex (no interaction).

c Limited to time from one year post-inclusion onwards, adjusted as Model 2 and stratified by sex (no interaction).

d Further limited to individuals with no diagnosis of aortic stenosis, atrial flutter, paroxysmal atrial fibrillation, hypertrophic cardiomyopathy, or perimyocarditis previously or within 6 months of inclusion and for the symptomatic population a left ventricular ejection fraction ≥40. Adjusted as Model 2 and stratified by sex (no interaction).

*P<0.001;

$ P<0.01;

§ P<0.05.

**Table 4:** Mean accumulated hospitalization time for cardiovascular and non-cardiovascular admissions, degree of CAD and sex in patients with angina compared to an asymptomatic reference population.

	Mean accumulated hospitalization time per 10 years of follow-up, days (95% confidence interval), age-adjusted to 60 years				
	Women	Men	Pooled data	Pooled data^a^	Pooled data^b^
Study population group	n = 8,050	n = 8,868	n = 16,918	n = 16,592	n = 10,115
**CV hospitalization**					
Reference	2.8(2.1–3.5)	3.9(3.1–4.7)	3.2(2.7–3.7)	3.5(3.0–4.0)	3.3(2.8–3.8)
Angiographically normal	3.5(2.9–4.2)	7.7(6.1–9.3)*	5.1(4.4–5.8)*	4.5(3.8–5.2)^§^	3.9(3.0–4.7)
Angiographically diffuse	7.6(4.5–10.8)^$^	8.6(6.5–10.7)*	7.9(6.1–9.8)*	7.0(5.4–8.6)*	7.1(4.0–10.1)^§^
1VD				6.7(5.2–8.1)*	6.6(4.2–8.9)^$^
2VD				6.1(4.9–7.4)*	5.3(4.0–6.6)^$^
3VD				8.6(6.6–10.7)*	8.3(6.3–10.3)*
**Non-CV hospitalization**					
Reference	16.6(15.2–18.1)	21.3(18.6–24.1)	18.9(17.5–20.4)	22.3(20.1–24.4)	22.1(19.9–24.2)
Angiographically normal	16.2(14.3–18.1)	25.2(21.0–29.5)	20.1(18.0–22.1)	20.6(18.3–22.8)	23.8(19.7–27.9)
Angiographically diffuse	25.8(15.8–35.8)	25.9(20.5–31.3)	25.8(20.2–31.3)^§^	21.1(17.4–24.8)	22.0(15.6–28.4)
1VD	18.5(14.8–22.2)	21.2(15.2–27.2)	19.7(15.6–23.7)	17.2(13.8–20.7)^§^	17.7(14.2–21.3)^§^
2VD	18.8(10.6–26.9)	20.0(15.7–24.2)	18.8(15.0–22.7)	18.0(14.0–22.0)	17.6(11.9–23.3)
3VD	24.0(15.6–32.4)	26.6(21.1–32.2)	25.3(20.6–30.0)^$^	21.4(17.3–25.5)	22.3(17.0–27.6)

VD: vessel disease. CAD: coronary artery disease. CV: cardiovascular.

*P≤0.001; ^$^P≤0.01; ^§^P≤0.05 for difference compared with the reference population.

a Limited to time from one year post-inclusion onwards.

b Sensitivity analysis further limited to individuals with no diagnosis of aortic stenosis, atrial flutter, paroxysmal atrial fibrillation, hypertrophic cardiomyopathy, or perimyocarditis previously or within 6 months of inclusion and for the symptomatic population a left ventricular ejection fraction ≥40.

### CAG

**Figure 3:**
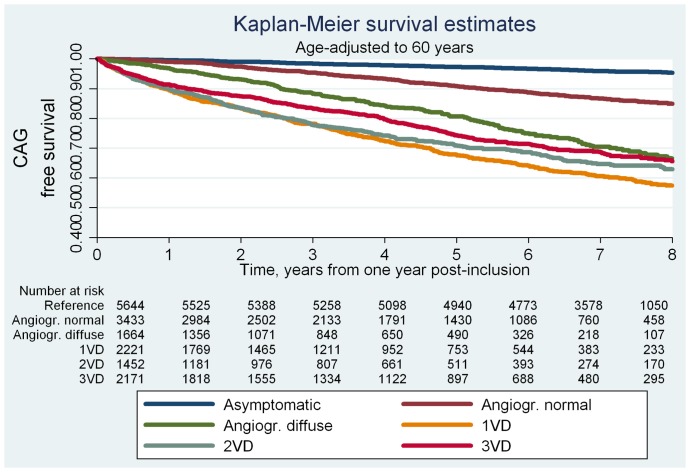
Kaplan Meier survival estimates for repeat CAG by study population group. CV: cardiovascular; CAG: coronary angiography; Angiogr.: angiographically; VD: vessel disease.

During follow-up, 1,101 women and 1,981 men had at least one additional CAG performed. In both men and women, patients with no obstructive CAD at angiography experienced higher rates of repeat CAG per 1000 years at risk (range 17–55) than the reference population (range 6–11) (Table 3). Hazard ratios were higher in women than in men, however, this pattern was not statistically significant (P for interaction with sex was 0.07). Pooling men and women yielded three- to fivefold increased risks of repeat CAG for patients with no obstructive CAD at baseline angiography compared with the reference population even after multivariable adjustment (Model 2, Table 4). Models limited to follow-up after one-year post-inclusion yielded multivariable adjusted HRs for patients with angiographically normal coronary arteries of 2.3(1.9–2.9, P<0.001), angiographically diffuse non-obstructive CAD of 5.5(4.4–6.8, P<0.001), and obstructive CAD of 6.6–9.4 (range, all P<0.001). The corresponding age-adjusted event-free survival curves for CAG are shown in Figure 3. The age-adjusted probability of CAG-free survival was lower in all patient groups compared with the reference population and similar in patients with angiographically diffuse non-obstructive CAD and patients with obstructive CAD.

### Non-cardiovascular Hospitalization

**Figure 4:**
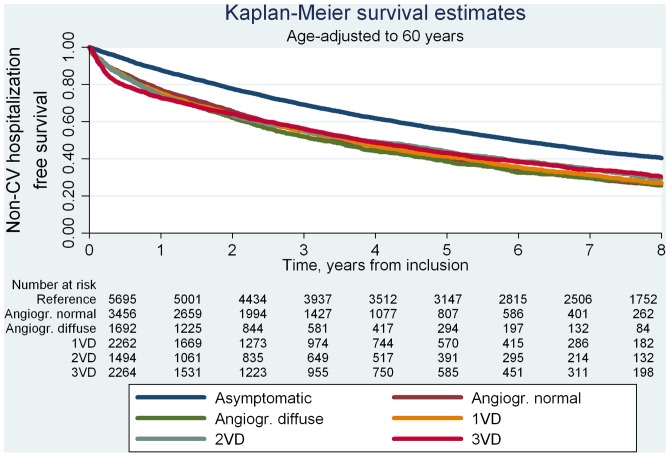
Kaplan Meier survival estimates for non-CV hospitalization by study population group. Non-CV: non-cardiovascular; Angiogr.: angiographically; VD: vessel disease.

During follow-up, 4,276 women and 4,407 men were hospitalized a total of 37,703 times due to non-cardiac causes. For both sexes, patients with no obstructive CAD at angiography showed higher rates of non-cardiovascular hospitalization per 1000 years of follow-up (range 396–499) than the reference population (range 249–250). We found no systematic sex differences in risk of non-cardiovascular hospitalization after age-adjustment (Model 1, Table 2). Thus, pooling men and women yielded multivariable adjusted HRs, for patients with angiographically normal coronary arteries of 2.0(1.8–2.1, P<0.001), angiographically diffuse non-obstructive CAD of 2.0(1.8–2.2, P<0.001) and obstructive CAD of 1.5–1.7(range, all P<0.001) compared with the reference population. Figure 4 shows non-cardiovascular hospitalization free survival curves. The probability of age-adjusted event-free survival was similarly low for all patient groups compared to the reference population.

Patients with angiographically diffuse non-obstructive CAD showed longer mean accumulated hospitalization times per 10 years of follow-up compared to the reference population and compared to patients with angiographically normal coronary arteries, 1VD and 2VD, respectively, (all, P<0.05) (Table 4, pooled analysis). Thus, we found a mean accumulated hospitalization time in days per 10 years of follow-up of 25.8(20.2–31.3) for patients with angiographically diffuse non-obstructive CAD and equal hospitalization times of 19–20(range) for reference individuals and patients with angiographically normal coronary arteries, 1VD and 2VD, respectively.

### Family Doctor Consultation

A total of 448,240 GP consultations occurred in the study population during follow-up. In both men and women, patients with no obstructive CAD at angiography experienced higher rates per 1000 years of follow-up of GP consultations (range 4600–5500) than the reference population (range 2700–3600). We found no systematic sex differences in risk of GP consultations after age-adjustment (Model 1, Table 3). Pooling men and women yielded multivariable adjusted HRs for patients with angiographically normal coronary arteries of 1.5(1.4–1.5, P<0.001), angiographically diffuse non-obstructive CAD of 1.5(1.4–1.5, P<0.001) and obstructive CAD (1VD–3VD) of 1.3–1.4(range, all P<0.001) compared to the reference population.

### Sensitivity Analyses

We tested the robustness of our results by limiting the analyses to individuals (patients and reference individuals) without cardiac comorbidity and to patients with a left ventricular ejection fraction of ≥40. With a decrease in risk of hospitalization for heart failure, this slightly lowered the estimated risk of cardiovascular hospitalization in patients with no obstructive CAD at angiography to HRs for patients with angiographically normal coronary arteries of 2.5(2.0–3.1, P<0.001) and for patients with angiographically diffuse non-obstructive CAD of 3.3(2.7–4.1, P<0.001) but otherwise did not affect our results (Table 2, model 4) and (Table S1, model 4). Sensitivity analyses adjusting for baseline comorbidity showed insignificant effects on our results, and standard time-to-first-event analyses yielded results comparable to those presented (data not shown).

## Discussion

This study demonstrates that in patients with symptoms of SAP and either angiographically normal coronary arteries or angiographically diffuse non-obstructive CAD, the risks of cardiovascular hospitalization and CAG are considerably larger than for asymptomatic reference individuals. For the first time, we report event rates and mean accumulated hospitalization times based on recurrent events analyses for a median follow-up of 7.8 years. Accordingly, risk of cardiovascular hospitalization in patients with SAP symptoms and no obstructive CAD at angiography were three- to fourfold higher than in the reference population and the related mean accumulated cardiovascular hospitalization times were 30–100% higher irrespective of cardiac risk factors and cardiac comorbidity. Importantly, risks of cardiovascular hospitalization and accumulated cardiovascular hospitalization times were similar in patients with diffuse non-obstructive CAD and patients with obstructive CAD in models ignoring the first year of follow-up, during which the obstructive-CAD patients were likely to receive invasive treatment. Risk of CAG was two-fold higher in patients with angiographically normal coronary arteries versus reference individuals and likewise, six-fold higher in patients with angiographically diffuse non-obstructive CAD which was only slightly lower than in patients with obstructive CAD. Regarding non-cardiovascular hospitalizations and GP consultations, patients with SAP symptoms generally showed higher event rates compared with the reference population while only minor differences were found between the patient groups. No systematic sex differences were found.

Prior studies have reported that angina with a normal CAG is associated with an excellent prognosis. [8], [9] However, small sample sizes and few events might have been an issue in these studies. In a large population, we have recently shown that patients with SAP symptoms and no obstructive CAD at angiography have increased risks of major cardiovascular events compared to an asymptomatic reference population. [1] This has also been highlighted in the WISE study. [10] The present study extends these findings by demonstrating an increased risk of overall cardiovascular hospitalization and repeat CAG in these patients.

### Angina and Cardiovascular Risk Despite No Obstructive CAD

Patients may have SAP symptoms and increased cardiovascular risk in the absence of obstructive CAD for several reasons. Some most likely have aortic stenosis, rhythm disturbances, hypertrophic cardiomyopathy or heart failure. However, after the exclusion of such patients from the present study, patients with no obstructive CAD at angiography still showed significantly increased risks of all outcomes. Some patients surely have angina-like symptoms due to non-cardiac causes like COPD, musculoskeletal disorders, gastric peptic diseases, personality or psychiatric disorders. [11] In the present study, adjustment for non-cardiac causes of angina-like symptoms (by adjustment for baseline comorbidity) had no effect on our results. Another possibility is that some patients with symptoms of SAP and no obstructive CAD at angiography have coronary microvessel dysfunction causing characteristic symptoms of cardiac ischemia. [12] Several studies have established high prevalence of abnormal coronary flow, abnormal coronary vasomotion and abnormal metabolic responses to stress consistent with myocardial ischemia in patients with no obstructive CAD.[13]–[15] Importantly, several studies have also demonstrated that coronary microvascular dysfunction is associated with increased risk of major adverse outcomes in patients with no obstructive CAD.[16]–[18] This corresponds well with the results from the present study and previous results showing that the adverse events associated with angina symptoms and no obstructive CAD at angiography is primarily of cardiovascular character. [1].

The increased total cardiovascular disease burden in patients with SAP symptoms and no obstructive CAD at angiography demonstrated in the present study is important, not only from the affected patients’ point of view but also from a socio-economic point of view. In 2006, based on data from the WISE study, Shaw et al. reported estimated average lifetime costs of about $800,000 for women with angina and no obstructive CAD at angiography and about $1,000,000 for women with obstructive CAD. [3] As nearly two thirds of women and one third of men examined with a first-time CAG due to symptoms of SAP have no obstructive CAD, the magnitude of economic resources allocated to the condition is obvious. All together, we believe that recognizing the disease burden associated with SAP symptoms and no obstructive CAD at angiography is an important step toward better management of the entity, including the subsets of patients suffering from coronary microvascular dysfunction who require more intensive cardiovascular care than routinely offered today.

### Strengths and Limitations

Our study reflects practice in a nation-wide sample of patients. The large sample size of 11,223 patients and 5,695 reference individuals, the wide age range, the 19,780 primary events and even higher numbers of secondary events during more than 7 years of follow-up and the near-complete follow-up (only 140 patients were excluded due to insufficient data) are important strengths of this study.

The study has some limitations. Detailed information on baseline comorbidity which might explain subsequent outcomes, was not recorded at inclusion. Instead, we collected data on disease specific hospitalizations and out-patient contacts by central registry linkage to allow for comorbidity adjustment of our models. Probably, the included patients’ angina symptoms were due to a variety of causes. However, adjustment for potential confounding cardiac and non-cardiac causes of angina symptoms had insignificant effect on the results.

All information was obtained from nationwide registries and the two databases containing information on the CAGs, all in which data entry is performed continuously. Thus the retrospective design and use of data in the present study are not likely to have introduced any bias. The data do not provide insight into the mechanisms of angina with no obstructive CAD at angiography and the increased risk of cardiovascular outcomes.

## Conclusions and Implications

In conclusion, our study shows that among patients referred for CAG due to symptoms of SAP, patients with angiographically normal coronary arteries and angiographically diffuse non-obstructive CAD have considerably higher cardiovascular disease burdens in terms of higher cardiovascular hospitalization rates, higher accumulated cardiovascular hospitalization times and higher re-catheterization rates compared to asymptomatic reference individuals. The cardiovascular hospitalization rates and accumulated cardiovascular hospitalization times in patients with angiographically diffuse non-obstructive CAD were as high as in patients with obstructive CAD when ignoring the first year post-CAG. Thus, contrary to common perception, excluding obstructive CAD by CAG does not ensure a low cardiovascular disease burden in patients with symptoms of SAP. Further risk stratification and treatment strategies targeting this group are warranted.

## Supporting Information

Appendix S1 **Data and data sources.**
(DOCX)

Table S1 **Event rates and hazard ratios (95% confidence intervals) by major cardiovascular end-point, study population group and sex in successively adjusted recurrent event models.**
(DOCX)
