# Supplementary material for: Burden of Hospital Admission and Repeat Angiography in Angina Pectoris Patients with and without Coronary Artery Disease: A Registry-Based Cohort Study
Source: PLoS One. 2014 Apr 4;9(4):e93170. doi: 10.1371/journal.pone.0093170 (PMC3976412; doi:10.1371/journal.pone.0093170)
Supplement: Appendix S1 — Data and data sources. (DOCX) [file pone.0093170.s001.docx]

## Supplemental appendix

Data and data sources

Danish residents have unique identification numbers that allows for linkage between national registers at the personal level. This ensures complete follow-up with respect to morbidity and mortality, data which was obtained from the Danish National Patient Registry containing registrations of all admissions to all Danish hospitals since 1977 according to the International Classification of Diseases, the 8th edition (ICD-8) until 1994 and the ICD-10 from 1994 onwards. The National Patient Registry has been validated and found well suited for epidemiological research.[1] The use of registry linkage, the ICD-coding and coding of revascularization procedures have been described in detail elsewhere.[2] Information on all GP consultations was obtained from The Danish Health Service Register holding information on all health services from all health contractors in primary health care since 1990.[3] Data on GP consultations was available on a monthly basis while all other data was registered by specific dates (all to March 2011). Information on cardiovascular risk factors was based on recordings at the time of CAG or inclusion in the CCHS. Data regarding the use of medication were obtained from the Danish Register of Medicinal Product Statistics (national prescription registry) which keeps records on all prescriptions dispensed from Danish pharmacies since 1995 according to the Anatomical Therapeutic Chemical (ATC) system.[4] Due to partial reimbursement of drug expenses by the healthcare system, pharmacies are obliged to register all dispensed prescriptions. This ensures a highly accurate register.

Reference List

(1) Andersen TF, Madsen M, Jorgensen J, Mellemkjoer L, Olsen JH. (1999) The Danish National Hospital Register. A valuable source of data for modern health sciences. Dan Med Bull 46: 263-268.

(2) Jespersen L, Hvelplund A, Abildstrom SZ, Pedersen F, Galatius S, et al. (2012) Stable angina pectoris with no obstructive coronary artery disease is associated with increased risks of major adverse cardiovascular events. European Heart Journal 33: 734-744.

(3) Sahl Andersen J, De Fine Olivarius N, Krasnik A. (2011) The Danish National Health Service Register. Scandinavian Journal of Public Health 39: 34-37.

(4) Gaist D, Sorensen HT, Hallas J. (1997) The Danish prescription registries. Dan Med Bull 44: 445-448.
