# Supplementary material for: Burden of Hospital Admission and Repeat Angiography in Angina Pectoris Patients with and without Coronary Artery Disease: A Registry-Based Cohort Study
Source: PLoS One. 2014 Apr 4;9(4):e93170. doi: 10.1371/journal.pone.0093170 (PMC3976412; doi:10.1371/journal.pone.0093170)
Supplement: Table S1 — Event rates and hazard ratios (95% confidence intervals) by major cardiovascular end-point, study population group and sex in successively adjusted recurrent event models. (DOCX) [file pone.0093170.s002.docx]

Table S1.

VD: vessel disease. MI: myocardial infarction.

^a^Adjusted for age.

^b^Adjusted for age, body mass index, diabetes, active smoking, use of antihypertensive medication and lipid lowering medication, respectively and stratified by sex (no interaction).

^c^Limited to time from one year post-inclusion onwards, adjusted as Model 2 and stratified by sex (no interaction).

^d^Further limited to individuals with no diagnosis of aortic stenosis, atrial flutter, paroxysmal atrial fibrillation, hypertrophic cardiomyopathy, or perimyocarditis previously or within 6 months of inclusion and for the symptomatic population a left ventricular ejection fraction ≥40. Adjusted as Model 2 and stratified by sex (no interaction).

‡P<0.05 indicates interaction with sex and estimates from these models should be interpreted with caution.

†Angina comprises the following ICD-10 codes: I20.1, I20.8, I20.9, I25.1 and I25.9.

*P<0.001; $P<0.01; §P<0.05; ‡P<0.05 indicates interaction with sex and estimates from these models should be interpreted with caution.

| Table S1. Event rates and hazard ratios (95% confidence intervals) by major cardiovascular end-point, study population group and sex in successively adjusted recurrent event models | | | | | | | |  |
| --- | --- | --- | --- | --- | --- | --- | --- | --- |
|  | Events; event rates per 1000 years of follow-up | | Model 1^a^ | | Model 2^b^ | Model 3^c^ | Model 4^d^ | |
| **End-point** | Women | Men | Women | Men | Pooled data | Pooled data | Pooled | |
| Study population group | n=8,050 | n=8,868 | n=8,050 | n=8,868 | n=16,918 | n=16,592 | n=10,115 | |
| **MI** |  |  |  |  |  |  |  | |
| Reference | 55; 2(2–3) | 79; 4(3–6) | (Reference) | (Reference) | (Reference) | (Reference) | (Reference) | |
| Angiographically normal | 24; 2(1–3) | 20; 3(2–5) | 1.3(0.7–2.2) | 0.7(0.4–1.2) | 0.8(0.5–1.2) | 0.8(0.5–1.2) | 0.7(0.4–1.3) | |
| Angiographically diffuse | 37; 9(6–14) | 49; 11(8–16) | 4.7(2.8–7.7)* | 2.3(1.5–3.5)* | 2.4(1.5–3.7)* | 2.9(2.0–4.3)* | 2.4(1.4–4.1)$ | |
| 1VD | 60; 13(10–20) | 113; 13(11–16) |  |  |  | 3.0(2.1–4.4)* | 2.5(1.5–4.0)* | |
| 2VD | 32; 14(10–22) | 93; 15(12–19) |  |  |  | 2.5(1.7–3.6)* | 1.9(1.1–3.0)§ | |
| 3VD | 54; 19(14–28) | 162; 15(13–19) |  |  |  | 2.3(1.5–3.4)* | 1.6(1.0–2.6)§ | |
| **Unstable angina** |  |  |  |  |  |  |  | |
| Reference | 23; 1(1–1) | 21; 1(1–2) | (Reference) | (Reference) | (Reference) | (Reference) | (Reference) | |
| Angiographically normal | 25; 2(1–3) | 21; 3(2–6) | 2.4(1.1–5.0)§ | 3.0(1.4–6.1)$ | 1.7(0.9–3.1) | 1.8(1.0–3.1)§ | 2.3(1.2–4.4)§ | |
| Angiographically diffuse | 34; 8(6–13) | 40; 9(7–13) | 9.7(4.7–19.7)* | 8.7(4.5–16.9)* | 4.9(2.6–9.2)* | 5.2(3.0–9.0)* | 5.2(2.8–10.0)* | |
| 1VD | 105; 23(18–30) | 166; 19(16–24) |  |  |  | 7.4(4.4–12.5)* | 5.8(3.1–10.7)* | |
| 2VD | 55; 24(17–36) | 129; 20(17–25) |  |  |  | 6.0(3.5–10.4)* | 5.1(2.7–9.6)* | |
| 3VD | 70; 25(18–36) | 155; 11(12–18) |  |  |  | 5.2(2.8–9.6)* | 4.7(2.5–9.1)* | |
| **Angina†** |  |  |  |  |  |  |  | |
| Reference | 69; 3(2–4) | 122; 7(5–8) | (Reference) | (Reference) | (Reference) | (Reference) | (Reference) | |
| Angiographically normal | 245; 19(14–27) | 148; 22(15–32) | 8.3(5.4–12.8)* | 3.4(2.3–5.3)* | 4.9(3.3–7.3)* | 4.5(3.2–6.5)* | 5.8(3.6–9.3)* | |
| Angiographically diffuse | 180; 45(36–57) | 231; 53(45–65) | 17.9(11.7–27.4)* | 7.4(5.4–10.2)* | 10.2(7.0–14.9)* | 9.7(7.2–13.1)* | 9.8(6.7–14.2)* | |
| 1VD | 1001; 224(206–244) | 1721; 201(188–215) |  |  | ‡ (P<0.01) | 18.5(13.9–24.6)* | 18.8(13.4–26.3)* | |
| 2VD | 544; 241(127–267) | 1485; 235(222–248) |  |  |  | 17.2(12.9–23.0)* | 17.4(12.3–24.4)* | |
| 3VD | 690; 248(226–273) | 2458; 233(223–244) |  |  |  | 15.3(11.3–20.7)* | 15.8(11.1–22.5)* | |
|  |  |  |  |  |  | ‡ (P<0.001) | ‡ (P<0.001) | |
| **Heart failure** |  |  |  |  |  |  |  | |
| Reference | 71; 3(2–4) | 65; 4(2–5) | (Reference) | (Reference) | (Reference) | (Reference) | (Reference) | |
| Angiographically normal | 83; 7(5–9) | 153; 23(14–38) | 3.6(2.3–5.6)* | 7.2(4.1–12.6)* | 6.0(3.9–9.2)* | 6.3(3.9–10.3)* | 3.2(1.6–6.0)$ | |
| Angiographically diffuse | 32; 8(5–15) | 108; 25(18–37) | 3.1(1.7–5.7)* | 6.1(3.7–10.2)* | 5.7(3.7–8.6)* | 6.2(3.9–9.8)* | 3.8(1.9–7.5)* | |
| 1VD | 46; 10(7–16) | 121; 14(10–20) | 4.1(2.5–6.8)* | 3.5(2.1–5.8)* | 4.3(3.0–6.3)* | 4.1(2.7–6.2)* | 2.1(1.1–4.2)* | |
| 2VD | 47; 21(14–33) | 88; 14(10–19) | 6.9(4.1–11.6)* | 3.2(2.0–5.1)* | 4.5(3.1–6.5)* | 4.6(3.0–6.9)* | 3.8(2.2–6.7)* | |
| 3VD | 42; 15(9–26) | 228; 21(17–27) | 4.2(2.4–7.4)* | 4.6(3.0–7.0)* | 5.2(3.6–7.4)* | 4.4(3.0–6.6)* | 3.1(1.7–5.9)* | |
|  |  |  |  |  | ‡ (P<0.01) | ‡ (P<0.01) | ‡ (P=0.4) | |
| **Stroke** |  |  |  |  |  |  |  | |
| Reference | 104; 4(3–5) | 67; 4(3–5) | (Reference) | (Reference) | (Reference) | (Reference) | (Reference) | |
| Angiographically normal | 62; 5(4–7) | 47; 7(5–10) | 1.6(1.1–2.3)§ | 2.1(1.4–3.2)* | 1.8(1.3–2.4)* | 1.7(1.2–2.3)$ | 2.2(1.5–3.3)* | |
| Angiographically diffuse | 35; 9(6–13) | 38; 9(6–13) | 2.0(1.3–3.2)$ | 2.1(1.3–3.3)$ | 1.9(1.4–2.7)* | 2.0(1.4–2.9)* | 2.0(1.2–3.2)$ | |
| 1VD | 31; 7(5–11) | 52; 6(5–8) | 1.6(1.0–2.6)§ | 1.5(1.0–2.2) | 1.4(1.0–2.0)§ | 1.4(0.9–2.0) | 1.5(1.0–2.2) | |
| 2VD | 12; 5(3–11) | 48; 8(6–10) | 1.0(0.5–2.0) | 1.7(1.2–2.6)$ | 1.4(1.0–2.0) | 1.3(0.9–1.9) | 1.4(0.9–2.1) | |
| 3VD | 27; 10(7–15) | 150; 14(12–17) | 1.7(1.1–2.7)§ | 3.0(2.2–4.2)* | 2.4(1.8–3.2)* | 2.1(1.5–2.8)* | 2.4(1.7–3.5)* | |
